# Preparation and Characterization of TCPP-CaMMT Nanocompound and Its Composite with Polypropylene

**DOI:** 10.3390/nano12091428

**Published:** 2022-04-22

**Authors:** Junming Geng, Yanhua Lan, Shanshan Liu, Jiyu He, Rongjie Yang, Dinghua Li

**Affiliations:** 1 School of Materials Science and Engineering, Beijing Institute of Technology, Beijing 100081, China; sscgjm@bit.edu.cn (J.G.); hejiyu@bit.edu.cn (J.H.); yrj@bit.edu.cn (R.Y.); 2 School of Environment and Safety Engineering, North University of China, Taiyuan 030051, China; yhlan@nuc.edu.cn; 3 Beijing Composite Materials Co., Ltd., Beijing 102101, China; 2120171326@bit.edu.cn

**Keywords:** tris-(1-chloropropan-2yl) phosphate, intercalated organic montmorillonite, polypropylene

## Abstract

Based on the molecular dynamics method, the tris-(1-chloropropan-2yl) phosphate (TCPP)/montmorillonite (MMT) molecular model was established to study the binding energy and microstructure changes in TCPP and MMT. The theoretical simulation results showed that TCPP can enter the MMT layer and increase the layer spacing. From this, an organic intercalated Ca-montmorillonite TCPP-CaMMT was prepared by a very simple direct mixing method using flame retardant TCPP as a modifier. Polypropylene (PP) composites were prepared by TCPP, CaMMT, and TCPP-CaMMT. The microstructures of TCPP-CaMMT nanocompounds and PP composites were studied by X-ray diffraction (XRD), scanning electron microscope (SEM), and transmission electron microscope (TEM). The results showed that TCPP-CaMMT nanocompounds could be exfoliated into nanosheets in PP. The flame retardancy and mechanical properties of PP/TCPP-CaMMT samples were studied by limited oxygen index (LOI) measurements and tensile tests. The PP/TCPP-CaMMT composites showed better LOI, tensile strength, and elongation at break than the machine-mixed PP/TCPP + CaMMT.

## 1. Introduction

Montmorillonite (MMT), as a natural material, is a very attractive nanosized polymer-reinforced filler because of its characteristic layered structure and large specific surface area [1,2]. The well-dispersed montmorillonite nanosheets can improve and enhance mechanical and flame retardant properties of polymer matrices [3,4]. However, unmodified montmorillonite, because of the presence of a large number of metal cations and hydroxyl hydrophilic groups in its crystal cells, shows a strong hydrophilia and poor compatibility with polymer matrices [5,6,7,8,9,10,11]. So, the particle size of MMT in a polymer is usually a micron.

After organic modification of montmorillonite, the compatibility between montmorillonite and polymer matrix is enhanced, which can effectively solve the agglomeration problem of natural montmorillonite in a polymer. The modifiers for organically modified montmorillonite (OMMT) include cationic modifiers, anionic modifiers, nonionic modifiers, zwitterionic modifiers, etc. Among these modifiers, the organic cation is the most common modifier, which can modify montmorillonite by cation exchange [12,13,14,15]. However, a significant problem with organic cation modification is that the amino group in the molecular structure has a negative effect on the thermal stability of montmorillonite. Therefore, in the past ten years, scholars have explored and prepared organic anionic montmorillonite, organic nonionic montmorillonite, organic zwitterionic montmorillonite, comodified montmorillonite, and their thermal stability was significantly improved compared with traditional organic cationic modifiers [16,17,18,19,20,21,22,23,24]. However, there are still many challenges in improving the flame retardancy of the polymer by modifying montmorillonite with the above organic modifiers. First, the commonly used organic modifiers have low flame retardancy efficiency as the organic modifiers themselves are flammable. Second, waste liquid is often produced in the modification process, which inevitably pollutes the environment to a certain extent.

Phosphorus-containing flame retardants have the advantages of high flame retardant efficiency, low toxicity, low corrosion, and good compatibility with polymer materials [25,26,27]. Montmorillonite modified with phosphorous flame retardant can improve the compatibility between montmorillonite and polymer matrix and have a synergistic flame retardant effect which has attracted widespread attention [28,29,30,31,32]. The ammonium polyphosphate (APP)-NaMMT nanocompounds were prepared by Yi et al. and applied to polypropylene (PP) composites [28]. The thermal stability and limited oxygen index (LOI) of the PP composites were improved by the APP-NaMMT nanocompounds. He et al. prepared 9,10-dihydro-9-oxa-10-phosphaphenanthrene-10-oxide (DOPO)-CaMMT by adding CaMMT into DOPO ethanol solution and then stirring and ultrasonicated at 80 °C for 4 h [29]. The layer spacing of CaMMT was expanded by DOPO to 2.5 nm from 1.5 nm. When the flame retardant DOPO-CaMMT (6 wt%) was added to the epoxy resin (EP), the peak heat release rate (p-HRR) of EP/DOPO-CaMMT was significantly lower than that of pure EP. However, the modification methods of MMT by flame retardants mentioned above, such as solvent method and melting method, are faced with various problems such as complex process, high cost, low intercalation efficiency, and solvent recovery, which is still far from the preparation goal of low cost, high productivity, and industrialization. Therefore, it is of great significance to explore more convenient modification methods by phosphorus-containing flame retardants without heating and solvents.

Recent advances in computational chemistry methods have led to a new understanding of clay interlayer structures and their interactions. Yang et al. [33] found that 3-chloro-2-hydroxypropyl trimethylammonium chloride (CHPTA) acted as an effective clay mineral hydration inhibitor by means of density functional theory (DFT). CHPTA was mainly adsorbed on MMT by hydrogen bonds and especially electrostatic force, and the presence of Na ions favors the adsorption of CHPTA on the 001 surface. Thus, chlorine-containing organic materials have a strong interaction with MMT. However, molecular dynamics simulation (MD) is a more simple and rapid method compared with DFT that could explain the experimental phenomenon qualitatively. Karatas et al. [34] studied the interaction characteristics of curcumin anticancer drug on MMT nanoparticles in the presence of amphiphilic polymer by the MD method. MMT has a high affinity towards either polymer or drug molecules, especially because of van der Walls interactions. 

In this paper, tris-(1-chloropropan-2yl) phosphate (TCPP) was selected to modify MMT directly based on the theoretical calculation. An intercalated TCPP-CaMMT nanocompound was prepared using a simple direct mixing method at room temperature without any other solvents. The structure, morphology, and thermal stability of TCPP-CaMMT were investigated by X-ray diffraction (XRD), scanning electron microscope (SEM), and thermogravimetric analysis (TGA). In order to study the dispersion of TCPP-CaMMT nanocomposites in the polymer, TCPP-CaMMT was added into the nonpolar crystalline resin PP. The effects of TCPP-CaMMT on flame retardancy and mechanical properties of PP composites were evaluated.

## 2. Materials and Methods

### 2.1. Materials

Ca-based montmorillonite (CaMMT) was purchased from Yazhuo Minerals Trading Co., Ltd., Shijiazhuang, China. Na-based montmorillonite (NaMMT) was obtained from Nanocor Inc. (Hoffman Estates, IL, USA). Tris-(1-chloropropan-2yl)-phosphate (TCPP), 98% (mixture of isomers) was commercially available from Shanghai Macklin Biochemical Co., Ltd., Shanghai, China. The co-polypropylene (PP) was from SK Grobal chemical, Seoul, Korea.

### 2.2. TCPP/CaMMT and TCPP/NaMMT Models

**Figure 1:**
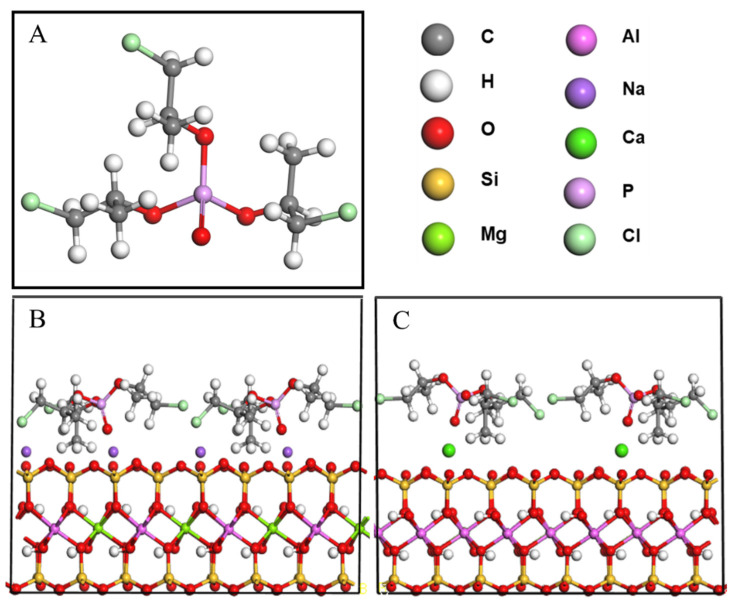
Molecular models of TCPP (**A**), TCPP/NaMMT (**B**), and TCPP/CaMMT (**C**).

The molecular structures of MMT and TCPP were established by the Visualizer module of the Materials Studio software package. CaMMT and NaMMT models were established according to cell parameters of Voora et al. [35], where lattice parameters a = 0.523 nm, b = 0.906 nm, c = 0.960 nm; α = γ = 90°, β = 99°, and the space group is C2/m, as shown in Figure 1. The (001) plane of MMT was cut, and the 2 × 2 supercell was processed. Four TCPP molecules were added onto the (001) plane of CaMMT and NaMMT, respectively, and a 2 nm vacuum layer was added to establish TCPP/CaMMT and TCPP/NaMMT models. In order to reduce the calculation time, the coordinates of MMT were fixed, and the TCPP/CaMMT and TCPP/NaMMT molecular models were optimized and simulated by the Forcite module. COMPASS II was used for the force field, and NVT ensemble was used for molecular dynamics. The temperature was set to 298 K, and the Nosé-Hoover-Langevin NHL method was adopted to control the temperature. The truncation radius was 0.95 nm, the time step was 1.0 ps, the total simulation time was 500 ps, and the output data were saved every 1000 steps. The last 50 ps data were used for subsequent property analysis, including binding energy and interface morphology.

### 2.3. Preparation of TCPP-MMT Nanocompound

TCPP and CaMMT were added to the mortar according to the mass ratio of 1:2 and 1:1. After grinding and mixing in the mortar for 5 min, the powder samples were obtained, which were named TCPP-CaMMT-1/2 and TCPP-CaMMT-1/1.

TCPP and CaMMT were added to the sample tube according to the mass ratio of 2:1, 3:1, 4:1, and 5:1. After evenly stirring, the gel samples were obtained, which were named TCPP-CaMMT-2/1, TCPP-CaMMT-3/1, TCPP-CaMMT-4/1, and TCPP-CaMMT-5/1.

TCPP and NaMMT were added into the sample tube according to the mass ratio of 2:1, stirred evenly, and let stand to obtain the gel sample, named TCPP-NaMMT-2/1.

### 2.4. Preparation of PP Composites

TCPP, CaMMT, TCPP + CaMMT, and TCPP-CaMMT-2/1 were fused, blended, extruded, and granulated on the twin-screw extruder (Brabender GmbH&Co. KG., Duisburg, Germany), according to the way of liquid and particle feeding, respectively, and then the specimens were obtained by injection molding. The final PP composites were individually named PP/TCPP, PP/CaMMT, PP/TCPP + CaMMT-2/1, and PP/TCPP-CaMMT-2/1. The total content of TCPP, CaMMT, TCPP + CaMMT, and TCPP-CaMMT-2/1 was 10 wt% in the PP, as shown in Table 1.

### 2.5. Measurements of PP Composites

#### 2.5.1. XRD Characterization

The X-ray diffraction (XRD) patterns of MMT, TCPP-MMT, and PP composites were conducted using a MiniFlex 600X-ray diffractometer with Cu Kα radiation (λ = 1.54178 Å). Operating conditions were 40 kV and 15 mA with a step size of 0.02°. The 2θ started at 2° and ended at 30°. Repeatability was tested by twice measuring every sample.

#### 2.5.2. SEM Characterization

An FEI Quanta 250 field-emission scanning electron microscope (FE-SEM) was employed to study the surface morphologies and elemental composition of the MMT, TCPP-MMT, and PP composites. The PP composites were sprayed with gold before testing.

#### 2.5.3. TEM Characterization

The morphologies of the PP composites were conducted by a Fei-Tecnai G2-F30 transmission electron microscope (TEM) instrument, operated at an acceleration voltage of 300 kV. PP composites need to be made into ultrathin slices before testing.

#### 2.5.4. TGA Characterization

Thermogravimetry analysis (TGA) was carried out using a Netzsch 209 F1 thermal analyzer in nitrogen. The heating rate was 10 °C/min, from 40 °C to 900 °C.

#### 2.5.5. LOI Analysis

The limited oxygen index test was carried out using the FTAII oxygen index meter (Rheometric Scientific LTD., Surrey, UK), according to the standard GB/T 2406.2. The dimensions of PP specimens were 120 mm × 6.5 mm × 3 mm.

#### 2.5.6. The Mechanical Properties Test

According to ISO 527-2 standard, the mechanical properties (tensile strength and elongation at break) of the PP composites were tested by CMT4101 microcomputer-controlled electronic universal testing machine at a tensile rate of 50 mm/min.

## 3. Results and Discussions

### 3.1. Intercalation Behavior in TCPP-MMT

**Figure 2:**
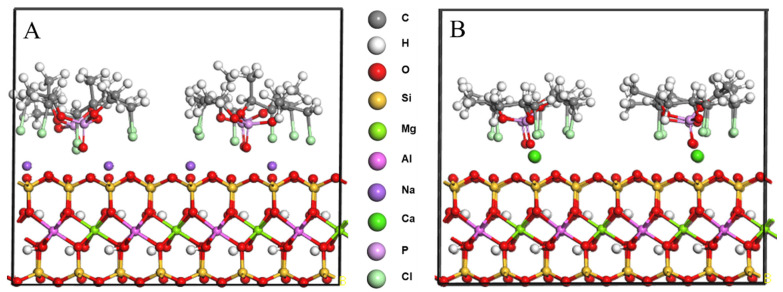
Molecular model after molecular dynamics. (**A**) TCPP/NaMMT; (**B**) TCPP/CaMMT.

In order to explore the feasibility of TCPP intercalating MMT, theoretical simulation was carried out at first. After molecular dynamics calculation, it was found that the microscopic morphology of the TCPP/MMT molecular model changed, as shown in Figure 2. The chlorine atom and P=O in TCPP pointed to MMT (001) crystal plane, indicating that TCPP and MMT had a strong polarity effect, resulting in the stable existence of TCPP between MMT layers.

The interactions of two materials include bond energy and nonbond energy, and nonbond interactions cover electrostatic attraction, van der Waals forces, and long-range force correction. For van der Waals forces, special treatment should be utilized to obtain the binding interactions between the Cl atom and other parts of the system by DFT approaches. Varadwaj [36] and Anderson [37] presented such a method of exchange and dispersion effect analysis with halogen-bonding interaction calculation to determine their equilibrium geometries, binding energies, and electronic properties. It is an indeed higher lever with better accuracy for the interaction calculation, while molecular simulation calculation needs less time with low accuracy. Here in our work, we have chosen the molecular dynamics method to calculate the interactions between MMT and small halogen-containing molecules aimed at a short computation time. The binding energies of TCPP with NaMMT and CaMMT are listed in Table 2. The binding energies of TCPP/NaMMT and TCPP/CaMMT systems all came from nonbonding interactions, including electrostatic interaction, van der Waals force, and long-range force correction, in which electrostatic interaction played almost 99% of the role. In order to eliminate the influence of model size on data, binding energy/area was given for comparison. The results show that the binding energy of calcium ion and TCPP is larger than that of sodium ion, indicating that the interaction force between calcium ion and TCPP is stronger.

**Figure 3:**
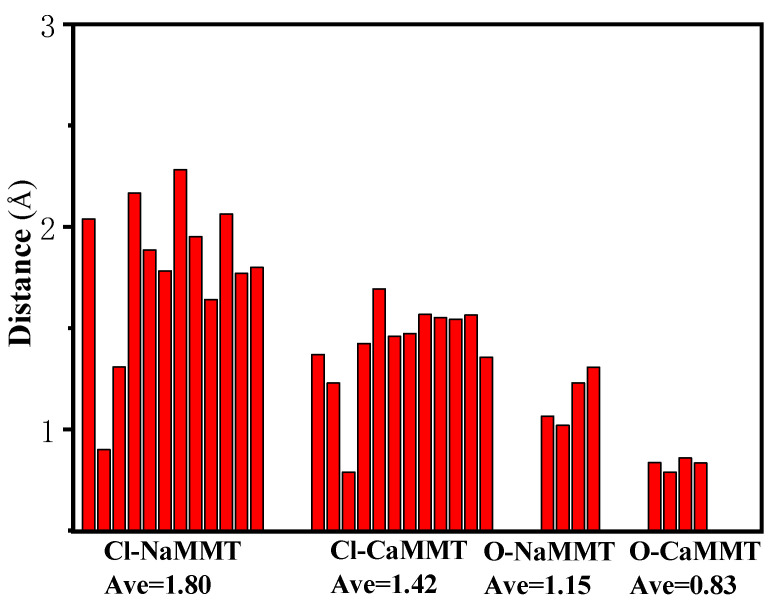
The distance between the chlorine atom and P=O to the surface of MMT.

**Figure 4:**
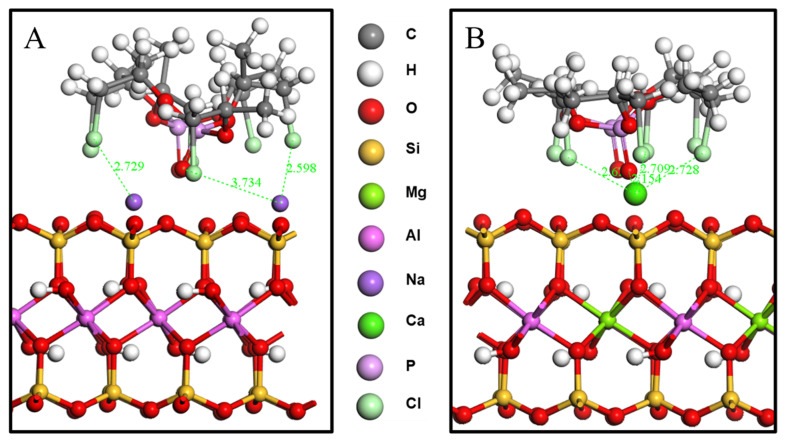
Close range annotation between molecules. (**A**) TCPP/NaMMT model; (**B**) TCPP/CaMMT model.

The distances between the main TCPP atoms (chlorine atoms and P=O) and the surfaces of the MMT were analyzed, as shown in Figure 3. In the system of TCPP and CaMMT, the average vertical distance between the chlorine atom and the plane where the Ca atom was located is 1.42 Å, and the average vertical distance between the O atom and the plane is 0.83 Å. In the system of TCPP and NaMMT, the average vertical distance between the chlorine atom and the plane where the Na atom is located is 1.80 Å, and the average vertical distance between the O atom and the plane is 1.15 Å, both of which are larger than CaMMT. The main reason for this phenomenon is that the binding energy of calcium ion and TCPP is greater than that of the Na ion and TCPP. Ca ion has a stronger interaction with TCPP, which could adsorb TCPP closer to MMT. Close range annotation between molecules in the model is shown in Figure 4.

Through theoretical simulation, we could preliminarily infer: (1) the polar organophosphate TCPP could enter the MMT layer and form the organic intercalated TCPP-MMT nanocompound; (2) the layer spacing in the TCPP-CaMMT was smaller than that in the TCPP-NaMMT.

**Figure 5:**
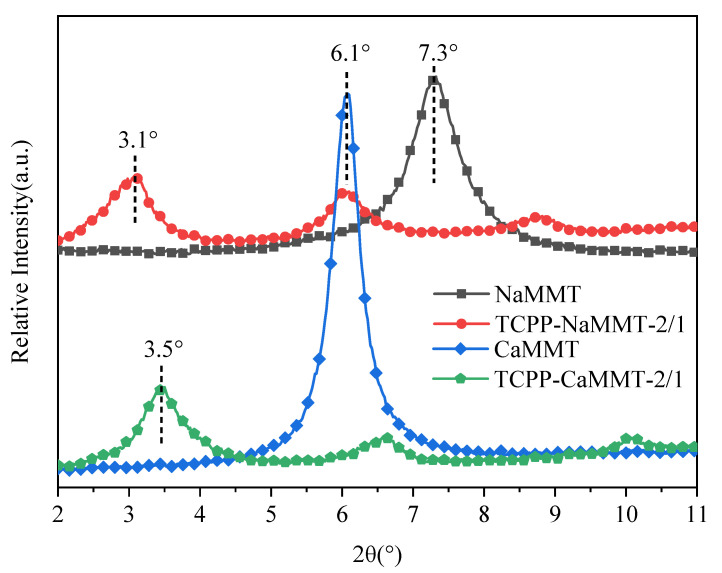
XRD pattern of TCPP/NaMMT and TCPP/CaMMT.

In order to verify the simulation results, TCPP-NaMMT-2/1 and TCPP-CaMMT-2/1 were prepared by the direct mixing method. The TCPP-NaMMT-2/1 and TCPP-CaMMT-2/1 were characterized by XRD, as shown in Figure 5.

The diffraction characteristic peak of NaMMT was located at 7.3°, corresponding to d_001_ = 1.2 nm. After TCPP was added to modify NaMMT, the diffraction peak of NaMMT moved to about 3.1°, and the d-spacing of TCPP-NaMMT-2/1 could be calculated as 2.8 nm. The diffraction characteristic peak of CaMMT was located at 6.1°, corresponding d001 = 1.4 nm. After TCPP was added to modify CaMMT, the diffraction peak of CaMMT moved to about 3.5°, and the d-spacing of TCPP-CaMMT-2/1 could be calculated as 2.5 nm. It indicated that TCPP could easily enter the interlamellar space of NaMMT and CaMMT and expand the interlayer spacing. The layer spacing of TCPP-CaMMT-2/1 nanocomposites was smaller than that of TCPP-NaMMT-2/1, which was consistent with the simulation results.

Considering that NaMMT is artificially Na-modification montmorillonite, that CaMMT is cheaper and more abundant in resources, and that calcium ions have a catalytic effect in combustion and pyrolysis, which could catalyze the formation of the carbon layer, the TCPP-CaMMT nanocompound would have a wider application value. Therefore we carried out further research on TCPP-CaMMT in the subsequent experiments.

### 3.2. Gelation of TCPP-CaMMT Nanocompound

**Figure 6:**
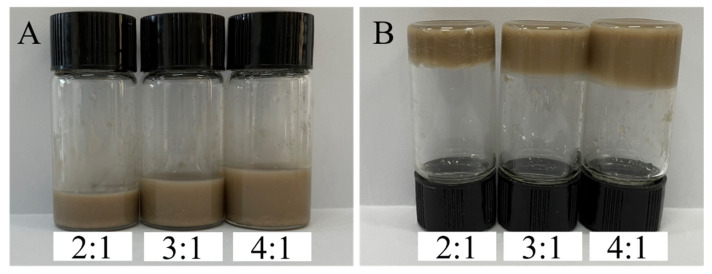
TCPP-CaMMT gel with different TCPP/CaMMT ratios. (**A**) before gel; (**B**) after gel.

The TCPP-CaMMT nanocompoud in different proportions were prepared after verifying that TCPP and CaMMT could obtain organic intercalated TCPP-CaMMT, and the gelation phenomenon of TCPP-CaMMT nanocompoud was analyzed. For the TCPP and CaMMT of the mass ratios 1:2 and 1:1, because of the small proportion of TCPP, CaMMT could not be fully contacted, and the mixture behaved as a sticky powder. For the TCPP and CaMMT of the mass ratios 2:1 and 4:1, the TCPP-CaMMT showed gelation and eventually formed a stable brown gelatinous complex, as shown in Figure 6. Because of that, when TCPP entered the layer spacing of MMT, the MMT layer spacing was enlarged. Under the action of TCPP solvation and stirring force, the montmorillonite crystal layer could be exfoliated and spontaneously dispersed to form a gel. For the sample TCPP to CaMMT as 5:1, the lower layer is viscous, and the upper layer is redundant TCPP liquid.

In order to evaluate the stability of TCPP-CaMMT gel under high stress, the TCPP-CaMMT samples with a mass ratio of 2:1 to 4:1 were centrifuged at high speed (12,000 r/min) for 30 min. TCPP liquid was observed in the upper layer of TCPP-CaMMT-3/1 and TCPP-CaMMT-4/1, as shown in Table 3. The gel state of TCPP-CaMMT-2/1 was relatively stable, and TCPP was not separated from the gel even under the condition of high centrifugal force.

### 3.3. XRD Patterns of TCPP-CaMMT Nanocompounds

**Figure 7:**
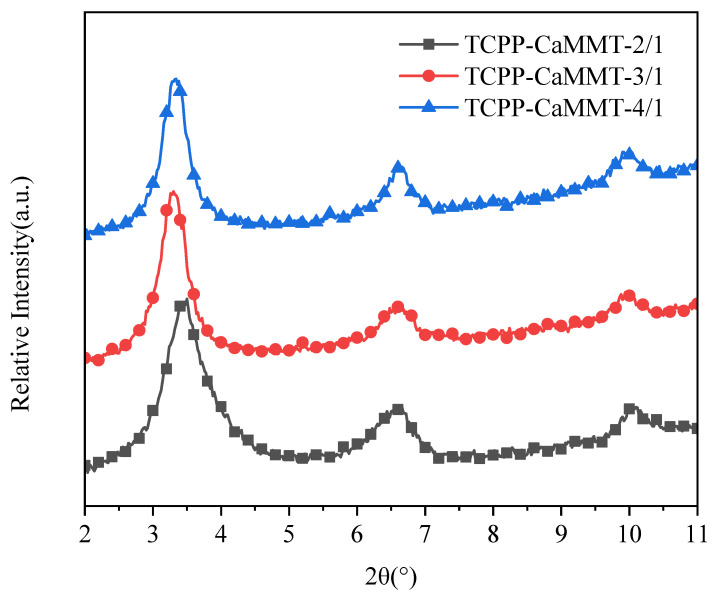
XRD patterns of TCPP-CaMMT nanocompounds.

We conducted XRD characterization of TCPP-CaMMT gels to investigate the influence of different amounts of TCPP on intercalation behavior, as shown in Figure 7. The 2θ values of TCPP-CaMMT nanogels of different mass ratios are basically similar (around 3.5°), indicating that the d-spacing of montmorillonite had little change in the TCPP-CaMMT gels of different mass ratios. In the TCPP-CaMMT-2/1, all of the TCPP is part of the stable gel network because of the intercalation. In the TCPP-CaMMT-3/1 and TCPP-CaMMT-4/1, the excess TCPP exists in the gel network without a strong physical interaction with the CaMMT layers, so it could be separated from the gel when subjected to high centrifugal force.

### 3.4. SEM Images of TCPP-CaMMT Nanocompounds

**Figure 8:**
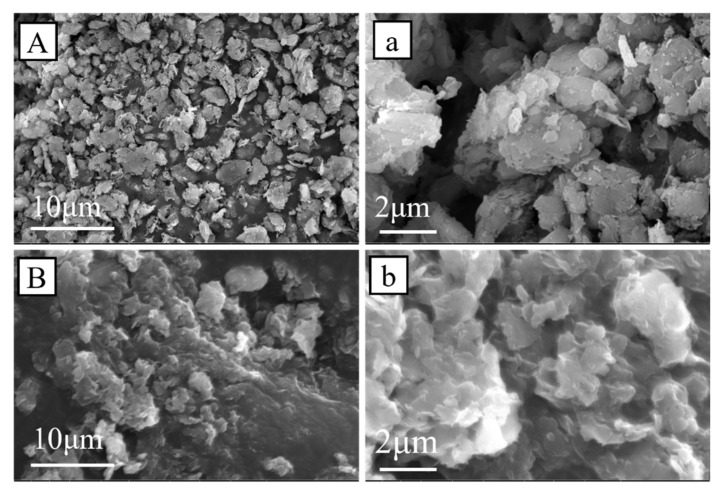
SEM images of CaMMT (**A**,**a**) and TCPP-CaMMT-2/1 (**B**,**b**).

The TCPP-CaMMT-2/1 and CaMMT were characterized by SEM, and their microscopic morphology was observed, as shown in Figure 8. The CaMMT mainly existed as agglomerated particles, whose sizes were mainly 3–7 μm, and the lamellar layers were closely stacked together. The TCPP-CaMMT-2/1 nanocompound was a gel block with a large amount of CaMMT aggregated together; at the surface, there was continuous lamellar floccule with soft edge contour, and some lamellar thickness was less than 100 nm. The results show that CaMMT modified by TCPP has loose intercalated and exfoliated structures.

### 3.5. Thermal Stability of TCPP-CaMMT Nanocompounds

**Figure 9:**
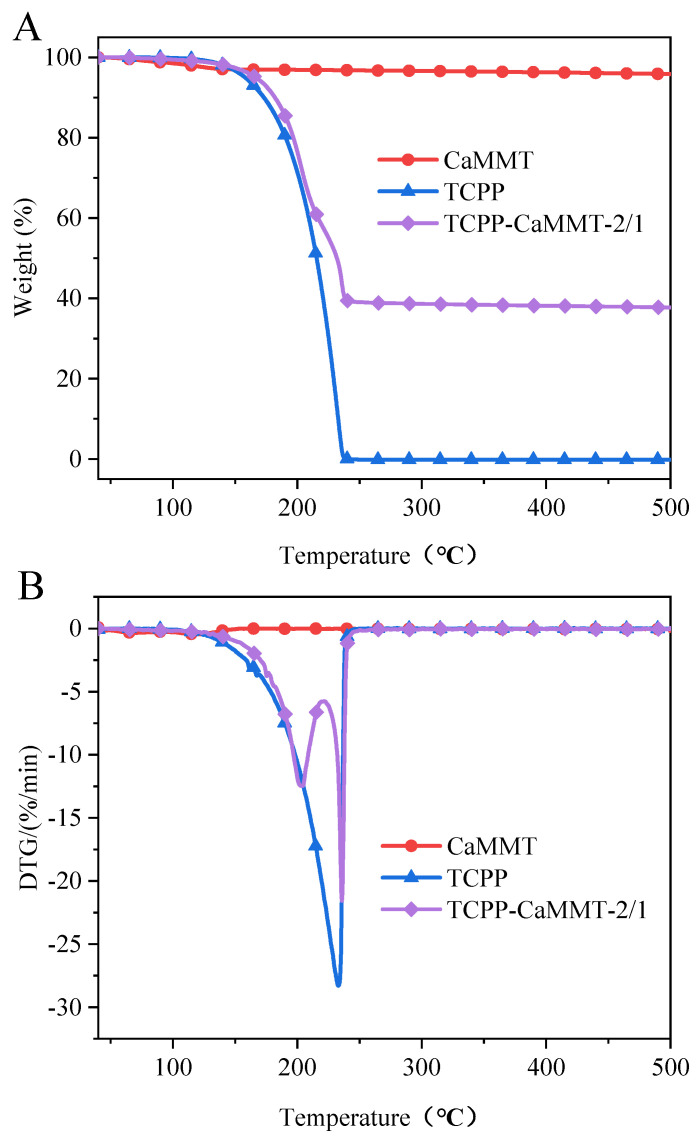
TG (**A**) and DTG (**B**) curves of TCPP-CaMMT-2/1 in N_2_.

TGA results of TCPP-CaMMT-2/1 and TCPP in N_2_ are shown in Table 4 and Figure 9. The thermal weight loss of TCPP starts at about 160 °C and ends at about 240 °C, and the temperature at the maximum weight loss rate, T_max_, is 233.0 °C. Compared with the rapid weight loss of TCPP in one stage, the thermal weight loss process of TCPP-CaMMT-2/1 nanocompounds could be divided into two stages; the T_max1_ and T_max2_ are 202.9 °C and 235.9 °C, respectively. The first stage is the weight loss of the free TCPP, and the second stage is the weight loss from TCPP intercalating into montmorillonite layers due to the strong interaction with montmorillonite and the thermal shielding of montmorillonite layers.

### 3.6. XRD Patterns of PP Composites

**Figure 10:**
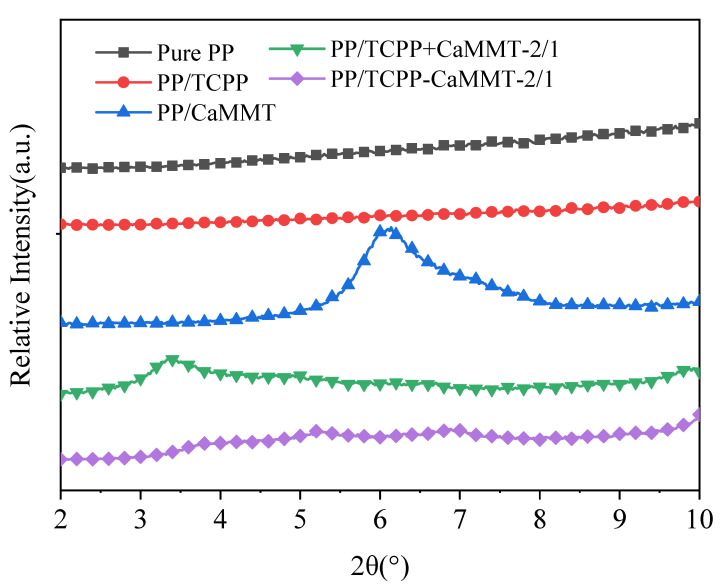
XRD patterns of PP composites.

The microstructures of montmorillonite in the PP matrix were investigated by XRD patterns, as shown in Figure 10. The characteristic peak of PP/CaMMT is at 6.1°, which is similar to that of natural CaMMT (d_001_= 1.4 nm), indicating that CaMMT still exists in a layered structure in the PP composites. The characteristic peak of PP/TCPP + CaMMT-2/1 appeared at 3.4°, indicating that the layer space of the montmorillonite had been increased in the PP/TCPP + CaMMT-2/1. This is because TCPP and CaMMT were in contact in the processing of PP composite, and the partial intercalation occurred between TCPP and CaMMT. However, the characteristic peaks of CaMMT, such as 3.4° and 6.1°, are not obvious in the XRD patterns of PP/TCPP-CaMMT-2/1, indicating that CaMMT exists in an exfoliated or amorphous state in the PP composite under the action of twin-screw shear force. The results show that the TCPP-CaMMT-2/1 sample had better dispersion in PP than the TCPP + CaMMT-2/1.

### 3.7. Microstructures of PP Composites

**Figure 11:**
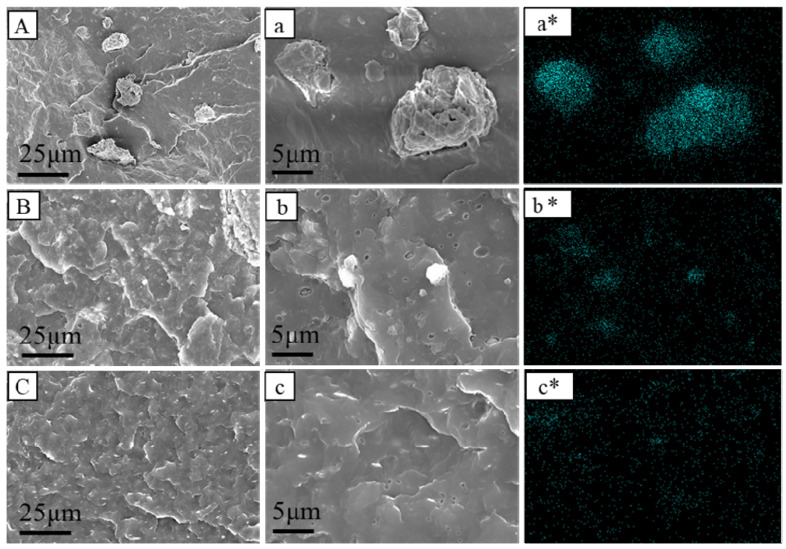
SEM diagram of PP composites. (**A**,**a**) PP/CaMMT; (**B**,**b**) PP/TCPP + CaMMT-2/1; (**C**,**c**) PP/TCPP-CaMMT-2/1; (a*–c*) Si distribution mapping.

The obtained PP/CaMMT, PP/TCPP + CaMMT-2/1, and PP/TCPP-CaMMT-2/1 composites were studied by SEM and TEM to further confirm the dispersion of CaMMT in the PP composites. The SEM images are shown in Figure 11. The raw CaMMT was aggregated in PP/CaMMT matrix with particle sizes concentrated in 5–20 microns in Figure 11, indicating that the compatibility between unmodified CaMMT and PP matrix is very poor. There are still some agglomeration particles in PP/TCPP + CaMMT-2/1 in Figure 11B,b. Compared with PP/CaMMT, the particle size of CaMMT in PP/TCPP + CaMMT-2/1 is reduced (generally less than 5 μm) because of the partial intercalation. The SEM image of PP/TCPP-CaMMT-2/1 (Figure 11C,c) exhibits a relatively smooth brittle section with almost no agglomeration particles, indicating that TCPP-CaMMT nanocompounds have good dispersion and compatibility in PP/TCPP-CaMMT-2/1. The mapping of Si distribution in Figure 11a*–c* further shows the distribution of montmorillonite intuitively. CaMMT is agglomerated in PP/CaMMT, and the corresponding silicon elements are also unevenly distributed. However, the Si element distribution on PP/TCPP-CaMMT-2/1 surface is relatively uniform, which indicates that TCPP-CaMMT-2/1 nanocompounds with large layer spacing can be dispersed evenly in the PP matrix.

**Figure 12:**
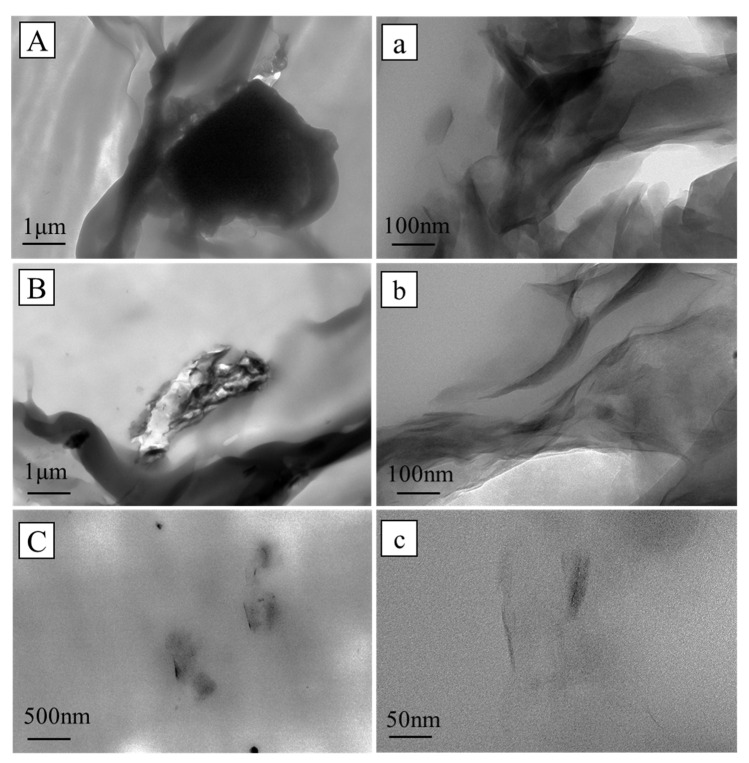
TEM image of PP composites. (**A**,**a**) PP/CaMMT; (**B**,**b**)PP/TCPP + CaMMT-2/1; (**C**,**c**) PP/TCPP-CaMMT-2/1.

The TEM images of CaMMT in the PP/CaMMT, PP/TCPP + CaMMT-2/1, and PP/TCPP-CaMMT-2/1 composites are illustrated in Figure 12. The raw CaMMT still exists in the form of a tightly stacked lamellar structure in PP/CaMMT with poor dispersion. CaMMT in PP/TCPP + CaMMT-2/1 mainly exists in lamellar stack particles, but in some areas, there are the intercalated and partially exfoliated CaMMT layers. CaMMT is uniformly dispersed in PP/TCPP-CaMMT-2/1 matrix in the form of nanosheets in Figure 12C,c. These results suggest that raw CaMMT has poor compatibility with the PP matrix and cannot be intercalated and exfoliated, while the organic intercalated TCPP-CaMMT nanocompounds have good compatibility and can be exfoliated under the sheer twin-screw force.

### 3.8. LOI Test and Tensile Test of PP Composites

The flame retardancy and mechanical properties of PP composites were studied by LOI test and tensile test. The results are shown in Table 5.

It could be seen from Table 5 that CaMMT had almost no significant contribution to PP, while TCPP could improve the LOI of PP and contribute to flame retardant performance. According to the LOI value of PP/TCPP-CaMMT-2/1 and PP/TCPP + CaMMT-2/1, the contribution of intercalated TCPP-CaMMT-2/1 to flame retardancy of PP was greater than that of machine-mixed TCPP + CaMMT-2/1. The results showed that the nano-dispersed montmorillonite had a better flame retardancy effect with TCPP. For the tensile test, the addition of TCPP and CaMMT reduced the tensile strength of PP, but the tensile strength of PP/TCPP-CaMMT-2/1 was higher than that of PP/TCPP, PP/CaMMT, and PP/TCPP + CaMMT-2/1, which indicated that uniformly dispersed montmorillonite nanosheets had a slowing effect on the decrease in the tensile strength of PP composites. The elongation at break of PP/TCPP-CaMMT-2/1 was the highest among the five PP composites, which was 130% and 36.8% higher than that of pure PP and PP/TCPP + CaMMT-2/1, respectively, indicating that the synergistic effect of nano-dispersed montmorillonite and TCPP was helpful to improve the fracture toughness of PP.

## 4. Conclusions

In this paper, TCPP was selected to modify montmorillonite directly based on the theoretical calculation that TCPP and CaMMT could interact with each other in a strong polarity and exist stably. The intercalated TCPP-CaMMT nanocompound was prepared using a simple direct mixing method at room temperature without other solvents. When the mass ratio of TCPP to CaMMT was 2:1 to 4:1, the TCPP-CaMMT nanocompound became gelatinous. In the PP/TCPP-CaMMT-2/1 composite, the TCPP-CaMMT was evenly dispersed in PP in the form of nanosheets. The LOI, tensile strength, and elongation at break of PP/TCPP-CaMMT-2/1 were better than those of PP/TCPP + CaMMT-2/1. It was mainly attributed to the nano dispersion of TCPP-CaMMT in PP and the synergistic effect of TCPP and nano-dispersed montmorillonite. TCPP-CaMMT nanocompound prepared by direct mixing method is simple, economical, and environmentally friendly and provides a new idea and opportunity for the industrialization of MMT modification.

## Figures and Tables

**Table 1:** Composition of PP composites.

Samples	PP	TCPP	CaMMT	TCPP-CaMMT-2/1
Pure PP wt%	100			
PP/TCPP wt%	90	10		
PP/CaMMT wt%	90		10	
PP/TCPP + CaMMT-2/1 wt%	90	6.667	3.333	
PP/TCPP-CaMMT-2/1 wt%	90			10

**Table 2:** Binding energy of TCPP with NaMMT and CaMMT.

Interaction Force	TCPP/NaMMT		TCPP/CaMMT	
	kcal/mol	kcal/mol/(Å^2^)	kcal/mol	kcal/mol/(Å^2^)
binding energy	1788.99	4.71	1820.33	4.80
non-bond interactions	1788.99	4.71	1820.33	4.80
van der Waals force	4.11	0.01	17.17	0.05
long-range force correction	3.82	0.01	3.82	0.01
electrostatic interaction	1781.06	4.69	1799.34	4.74

**Table 3:** Phase separation of TCPP-CaMMT gel with different TCPP/CaMMT ratios.

Samples	Compositions	Phase Separation	Separated TCPP Liquid/g
TCPP-CaMMT-2/1	TCPP 2 g/CaMMT 1 g	NO	0
TCPP-CaMMT-3/1	TCPP 3 g/CaMMT 1 g	YES	0.27
TCPP-CaMMT-4/1	TCPP 4 g/CaMMT 1 g	YES	1.19

**Table 4:** Thermogravimetric data of TCPP-CaMMT-2/1 in N_2_.

Samples	Temperature/°C			Residual Amount at 500 °C/%
	T_onset_	T_Max1_	T_Max2_	
TCPP	158.0	233.0	-	0.17
TCPP-CaMMT-2/1	166.5	202.9	235.9	37.72

**Table 5:** Flame retardancy and mechanical properties of PP composites.

Samples	Pure PP	PP/TCPP	PP/CaMMT	PP/TCPP + CaMMT-2/1	PP/TCPP-CaMMT-2/1
LOI (%)	18.2	21.2	18.1	18.7	19.0
Tensile strength(MPa)	31.4 ± 0.4	24.9 ± 0.5	27.9 ± 0.4	25.4 ± 0.6	26.5 ± 0.5
Elongation at break(%)	7.9 ± 0.3	8.6 ± 0.5	8.9 ± 0.4	13.3 ± 0.5	18.2 ± 0.3

## Data Availability

The data presented in this study are available upon request from the corresponding author.
